# Effect of Ataluren on dystrophin mutations

**DOI:** 10.1111/jcmm.15319

**Published:** 2020-04-28

**Authors:** Joachim Berger, Mei Li, Silke Berger, Michelle Meilak, Jeanette Rientjes, Peter D. Currie

**Affiliations:** ^1^ Australian Regenerative Medicine Institute Monash University Clayton Vic Australia; ^2^ Victoria Node EMBL Australia Clayton Vic Australia; ^3^ Monash Genome Modification Platform Monash University Clayton Vic Australia

**Keywords:** Ataluren, *dmd*, Duchenne muscular dystrophy, dystrophin, muscle, PTC124, zebrafish

## Abstract

Duchenne muscular dystrophy is a severe muscle wasting disease caused by mutations in the dystrophin gene (*dmd*). Ataluren has been approved by the European Medicines Agency for treatment of Duchenne muscular dystrophy. Ataluren has been reported to promote ribosomal read‐through of premature stop codons, leading to restoration of full‐length dystrophin protein. However, the mechanism of Ataluren action has not been fully described. To evaluate the efficacy of Ataluren on all three premature stop codons featuring different termination strengths (UAA > UAG > UGA), novel dystrophin‐deficient zebrafish were generated. Pathological assessment of the muscle by birefringence quantification, a tool to directly measure muscle integrity, did not reveal a significant effect of Ataluren on any of the analysed dystrophin‐deficient mutants at 3 days after fertilization. Functional analysis of the musculature at 6 days after fertilization by direct measurement of the generated force revealed a significant improvement by Ataluren only for the UAA‐carrying mutant *dmd^ta222a^*. Interestingly however, all other analysed dystrophin‐deficient mutants were not affected by Ataluren, including the *dmd^pc3^* and *dmd^pc2^* mutants that harbour weaker premature stop codons UAG and UGA, respectively. These in vivo results contradict reported in vitro data on Ataluren efficacy, suggesting that Ataluren might not promote read‐through of premature stop codons. In addition, Ataluren had no effect on dystrophin transcript levels, but mild adverse effects on wild‐type larvae were identified. Further assessment of N‐terminally truncated dystrophin opened the possibility of Ataluren promoting alternative translation codons within dystrophin, thereby potentially shifting the patient cohort applicable for Ataluren.

## 1 INTRODUCTION

Duchenne MD and Becker MD are two types of inherited muscular dystrophy (MD) that are caused by mutations within the dystrophin (*DMD*) gene. In the more severe form Duchenne MD, patients experience progressive deterioration of muscle strength, wheelchair dependency and ultimately early death usually due to respiratory or cardiac failure. The current standard of Duchenne MD treatment is the corticosteroid prednisone (prednisolone), which only mitigates the rate of muscle degeneration.^1^ In addition, the exon‐skipping drug Eteplirsen has been approved by the Food and Drug Administration of the USA (FDA) but not the European Medicines Agency (EMA).^2^

In 2007, Ataluren (Translarna, PTC124) was identified in a high‐throughput small molecule screen and suggested to promote suppression of nonsense mutations.^3^ In cell culture experiments, the efficiency of Ataluren was reported to inversely correlate with termination efficiencies in vitro; being most efficient with UGA, the most permissive PTC, followed by UAG and then UAA.^3^ In addition, oral administration of Ataluren ameliorated the dystrophic condition of the *mdx* mouse, which harbours the nonsense mutation UAA in *Dmd* that is least susceptible to Ataluren.^3^ Importantly, these findings were questioned by later reports that demonstrated that Ataluren stabilized the firefly luciferase reporter used to identify Ataluren.^4^, ^5^

PTCs within dystrophin have been identified as the genetic cause of Duchenne MD in approximately 10%‐15% of patients.^6^, ^7^ In small‐scale as well as in randomized, double‐blind, placebo‐controlled studies, Ataluren was found to have beneficial effects on disease progression in patients carrying nonsense mutations within dystrophin.^8^, ^9^, ^10^, ^11^ However, Ataluren treatment showed a significant clinical benefit only in the subgroup of patients with a baseline 6‐minute walk distance of 300 m or < 400 m, no significant effect was recorded for other subgroups.^10^ In addition, in studies of Duchenne muscular dystrophy patients, the nonsense mutation type was found to be not associated with beneficial effects of Ataluren.^11^, ^12^ These studies also revealed that Ataluren was generally well tolerated and most adverse clinical events were mild to moderate in severity,^10^, ^12^ including a possible reduction in body mass index.^8^ Therefore, the discussion about Ataluren efficacy on Duchenne MD patients with nonsense mutations as well as the mechanism of Ataluren function has not been resolved.^13^ As a result, Ataluren has been recently approved by the EMA but not the FDA.

Zebrafish are a valuable model to study Duchenne MD due to their close replication of the human condition,^14^ as well as their fecundity and genetic susceptibility that enables rigorous phenotypic evaluation. In addition, robust assays have been established that allow quantification of muscle parameters within zebrafish larvae. The birefringence assay employs the muscle birefringence, a light effect provoked by the diffraction of polarized light through the pseudo‐crystalline array of the muscle myofibril, causing muscle fibres to appear bright in an otherwise dark environment.^15^ Therefore, dystrophic muscle of dystrophin‐deficient mutants that feature stochastic myofibre breakdown display a patchy birefringence pattern and their phenotypic severity can be quantified at 3 days after fertilization (dpf). A further assay directly measures the force generated by whole mounted zebrafish larvae,^16^ thereby enabling quantification of muscle parameters in zebrafish models of muscle diseases.^17^, ^18^ Importantly, Ataluren administration to the dystrophin‐deficient *dmd^ta222a^* zebrafish significantly ameliorated the force generation of homozygous larvae and restored low levels of dystrophin protein.^19^

In this study, additional zebrafish dystrophin‐deficient lines were generated to enable evaluation of Ataluren efficiency in relation to different PTCs. In an extensive series of Ataluren treatments, only *dmd^ta222a^* homozygotes were ameliorated by Ataluren. Interestingly, the TAA nonsense mutation within *dmd^ta222a^* has been reported to be least effective for Ataluren treatment.^3^ The other tested mutants carrying TAG and TGA nonsense mutations or a frame‐shifting deletion remained unaffected, indicating that Ataluren might not function by ribosomal PTC read‐through. In addition, Ataluren had mild adverse effects on wild‐type (WT) larvae, which is consistent with human trials.^8^, ^10^ Digital droplet PCR revealed that Ataluren had no effect on dystrophin transcript in *dmd^ta222a^* mutants and WT siblings, indicating that the ameliorative effect of Ataluren was not conveyed via dystrophin transcript. In contrast to the other mutants that carry downstream mutations (*dmd^pc2^* in exon 32, *dmd^pc3^* in exon 34), the mutation of *dmd^ta222a^* locates to exon 4. Transgenic assessment of dystrophin protein showed that N‐terminally truncated dystrophin protein lacking exons 1‐7 was able to partially rescue the dystrophic phenotype of dystrophin‐deficient zebrafish. This opens the possibility that, instead of supressing PTC, Ataluren might promote translation of dystrophin from alternative start codons. However, further insights into Ataluren function are required to reveal its mechanism of action and to identify the patient cohort responsive to Ataluren treatment.

## 2 MATERIAL AND METHODS

### 2.1 Generation and genotyping of zebrafish mutant lines

A total of 48 males were mutagenized with N‐ethyl‐N‐nitrosourea (ENU) as described before^20^ and approved by the Monash Animal Service (MAS/2009/05). Surviving fish were outcrossed, and obtained F1 fish were crossed to heterozygous *dmd^ta222a^*. Resulting offspring was analysed for complementation via the birefringence assay at 3 days after fertilization (dpf). This non‐complementation screen identified the *dmd^pc3^* dystrophin allele, which was subsequently genotyped by PCR with the primers cDMD_DdeI_F (5′‐ccgctcatcggtagagggtatgccaagtgctt) and gDMD_DdeI_R (5′‐gagcactacaatcagtgaatgataacaa) followed by restriction digestion with DdeI (NEB).

To generate a frame‐shift mutation within dystrophin, the CRISPR/Cas9 technology was utilized following established methods.^21^ Briefly, two crRNAs (targeting 5′‐aggccaaaatgcagatgtcaAGG and 5′‐aaagttgctgcaatccgataAGG), tracrRNA and Cas9 (IDT) were simultaneously micro‐injected into zebrafish eggs. Resulting mosaic fish were identified by PCR with the primers cDMD71F (5′‐gcgattggttggaggcca) and cDMD77R (5′‐ttgcttgtgtcgtcgtctgc) and outcrossed for germline transmission, resulting in the *dmd^−69bp^* mutant line.

Founder mutants of *dmd^pc3^* and *dmd^−69bp^* were backcrossed to TU over 6 generations before experiments were performed. Animal breeding was approved by MAS/2009/02BC and MARP/2015/004/BC.

### 2.2 Ataluren treatment

Ataluren (Selleck Chemicals) was dissolved as a stock solution of 10 mmol/L in Dimethyl sulfoxide (DMSO) and in experiments directly added to the fish water at a final concentration 0.5 µmol/L. The Ataluren concentration of 0.5 µmol/L was established as optimal effective concentration within zebrafish previously.^19^ The control group was treated with 0.005% DMSO as a negative vehicle control, thereby matching the DMSO concentration used with Ataluren‐treated fish. Zebrafish embryos were dechorionated at 24 hpf before treatment. Fish water solutions were exchanged on a daily basis until analysis.

### 2.3 Quantification of birefringence

At 72 hpf, individual zebrafish larvae were automatically imaged in an unbiased way using the Abrio LS2.2 microscope as previously described.^15^ To maximize uniformity of larvae stages, all larvae of an analysed clutch were kept at 25°C during the imaging process in order to slow down larvae development without adverse effects.^22^ In addition, all imaging was performed within less than 1 hour. Subsequently, the first 20 somites of imaged larvae were selected using the software ImageJ and the mean of all grey values of the pixels was measured. To enable comparison of the birefringence from different larvae, obtained grey values were rescaled to control siblings set to 100%. To rescale values of siblings, measured grey values (*A*_1_ to *A_n_*) of each larva were multiplied by 100 and divided by the average of all measured grey values of siblings using $\frac{A_{i}\times 100}{\sum_{i=1}^{n}A_{i}/n}$ . To normalize values of mutants, measured grey values (*B*_1_ to *B_n_*) of each larva were multiplied by 100 and divided by the average of the measured grey values of the siblings using $\frac{B_{i}\times 100}{\sum_{i=1}^{n}A_{i}/n}$ . From six independent clutches, a minimum of five siblings and five mutants were analysed for their muscle birefringence, each treated with either 0.005% DMSO (negative vehicle control) or 0.5 µmol/L Ataluren as indicated. Finally, all analysed animals were genotyped by PCR as described above.

### 2.4 Statistical analysis

Statistical significance was calculated using the software Prism (GraphPad Software). Between two groups, significance was determined by Student's *t* test and for multiple groups one‐way ANOVA with post hoc Tukey's test was used. Presented data are mean ± SEM, calculated utilizing error propagation.

### 2.5 Force measurement

6‐dpf‐old larval were individually mounted at slack length between a force transducer and a puller with aluminium clips as described earlier.^23^ In short, whole larvae preparations were kept at 22°C in physiological buffered solution and stimulated through electrical pulses of 0.5 ms duration (supramaximal voltage) to give single twitches. Isometric force analysis was performed at various larva lengths to identify the optimal muscle length for maximal active force quantification. Single twitches were separated in 2‐min intervals and performed at stepwise‐increased length. At each length, active contraction was recorded to identify the maximal active force at optimal length. Subsequent to analyses, all animals were genotyped by PCR as described above.

### 2.6 Generation of transgenic zebrafish lines

RT‐PCRs were performed on total RNA isolated from 3‐dpf‐old wild‐type larvae to generate partial dystrophin cDNA constructs, which were combined using the InFusion system (Clontech). The assembled cDNA encoded for dystrophin A0A0R4IXX0 (UniProt) missing P3531 and Q3532 of exon 76. According to previous dystrophin cloning results,^24^ exon 78 (ENSDARE00001210285) was found to be non‐coding due to insertion of an additional exon between the annotated exons 77 (ENSDARE00001123314) and 78, resulting in GGRLNP at the C‐terminal end of dystrophin. The eGFP open reading frame was added at the 3′ end to the dystrophin cDNA, which was then placed into pDONR221 of the Gateway cloning system (Invitrogen) resulting in pME‐dmdGFP. pME‐dmd^∆ex1‐7^GFP was prepared by the InFusion PCR cloning system (Clontech) using pME‐dmdGFP as template and the primers cDMD55F (5′‐gcaggctatgttgctacagtccatccaga‐3′) and cDMD66R (5′‐agcaacatagcctgcttttttgtacaaagtt‐3′). Both cloned plasmids were combined with p5E‐acta1, p3E‐polyA and pDestTol2pACryGFP to generate pcryGFP‐acta1‐dmdGFP and pcryGFP‐acta1‐dmd^∆ex1‐7^GFP.^25^, ^26^, ^27^ The resulting plasmids were microinjected into wild‐type 1‐cell stage embryos to generate the transgenic lines *Tg(cry:GFP,acta1:dmdGFP)* and *Tg(cry:GFP,acta1:dmd^∆ex1‐7^GFP)*. Animal experiments were approved by IBC/22219.

### 2.7 Digital droplet PCR

Total RNA was isolated from two 6‐dpf‐old pooled larvae using TRI‐Reagent (Merck), and the Direct‐zol RNA kit (Zymo‐Research). ddPCR was performed using the QX200 Droplet Digital PCR Platform according to the manufacturer (Bio‐Rad). In short, the ddPCR reaction was comprised of 1 × Supermix, 15 mmol/L dithiothreitol, 440 U reverse transcriptase, 40 ng of total RNA template, 900 nmol/L of each forward primer (targeting *dmd* or *polr2d*), 900 nmol/L of each reverse primer (targeting *dmd* or *polr2d*), 250 nmol/L of each double‐quenched probe (FAM or HEX labelled) and nuclease‐free water to a total of 22 µL. Probe and primer sequences were (IDT): Forward primer targeting exon 38 of *dmd* (5′‐aagatttcctggaggatgcg); reverse primer targeting exon 40 of *dmd* (5′‐tgtcatggagaaggttgtgt); *dmd* probe (5′‐6FAM‐cccggtgga‐ZEN‐gagaaacgagaggccg‐3IABkFQ); forward primer targeting exon 1 of *polr2d* (5′‐gtcccagctcatgtttccta); reverse primer targeting exon 2 of *polr2d* (5′‐ttcatgaagacctcggacag); *polr2d* probe (5′‐HEX‐cctccgcgc‐ZEN‐tctcgttctgctgc‐3IABkFQ). Subsequently, 70 µL droplet generation oil (Bio‐Rad) was added to the ddPCR reaction and the Bio‐Rad QX200 droplet generator (Bio‐Rad) was utilized to generate up to 20 000 droplets from each sample. After PCR amplification, the QX200 Droplet Reader (Bio‐Rad) was used for automatic readout and results were analysed with the QuantaSoft software (Bio‐Rad).

## 3 RESULTS

### 3.1 Generation of novel dystrophin‐deficient zebrafish mutants

**FIGURE 1:**
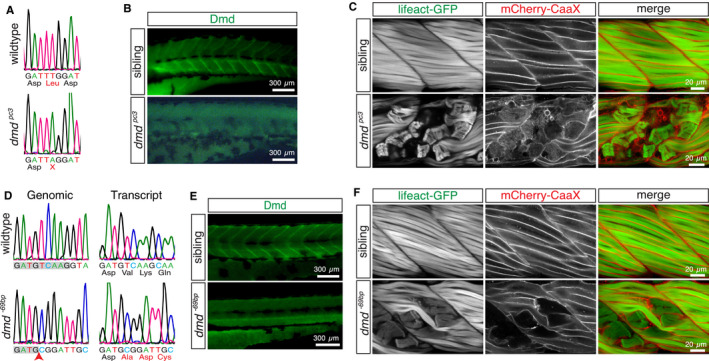
Novel dystrophin‐deficient mutants. A, A > T substitution within exon 34 of *dmd^pc3^* results in a nonsense mutation. B, Whole‐mount immunohistochemistry with antibodies against dystrophin revealed loss of dystrophin protein in 3‐dpf‐old *dmd^pc3^* homozygotes. C, At 3 dpf, labelling of the sarcolemma with mCherryCaaX and the myofibril with Lifeact‐GFP confirmed myofibre detachment within *dmd^pc3^* homozygotes. D, 5 bp from exon 53 and 64 bp from the downstream intron was removed from the dystrophin gene in *dmd^−69bp^* mutants. Altered splicing in *dmd^−69bp^* led to integration of 10 bp from the intron downstream of exon 53 into the dystrophin transcript, resulting in a frameshift and multiple subsequent PTCs. E, Dystrophin protein is lost in *dmd^−69bp^* homozygotes, as indicated by antibodies against dystrophin at 3 dpf. F, Retracting myofibres within 3‐dpf‐old *dmd^−69bp^* were revealed in the double transgenic background of *Tg(acta1:mCherryCaaX)* and *Tg(acta1:lifeact‐GFP)*

The dystrophin‐deficient zebrafish mutant line *dmd^ta222a^* harbours a nonsense mutation in exon 7 that encodes the PTC UAA^28^ and *dmd^pc2^* carry UGA in exon 32.^20^ To assess the reported inverse correlation of Ataluren efficacy with termination efficiencies of the three different PTCs in an animal model of Duchenne MD, novel zebrafish mutants were generated. In a genetic non‐complementation screen, male zebrafish were outcrossed after ENU treatment and resulting F1 founders were crossed to heterozygous *dmd^ta222a/+^* fish. The resulting offspring was assessed for complementation of the *dmd^ta222a^* allele by birefringence analysis at 3 dpf. This genetic non‐complementation approach resulted in the identification of a novel dystrophin mutant, *dmd^pc3^*, that harboured an in‐frame PTC within exon 34 (Figure 1A) leading to loss of dystrophin protein (Figure 1B). Myofibre detachment, typical for dystrophic muscle, was demonstrated within *dmd^pc3^* in the transgenic background of *Tg(acta1:mCherryCaaX)* and *Tg(acta1:lifeact‐GFP)*, in which mCherryCaaX highlights the sarcolemma together with t‐tubules and Lifeact‐GFP directly marks actin thin filaments (Figure 1C).^29^

To generate a dystrophin mutant with a frameshift allele, which should not be affected by PTC suppression, the CRISPR/Cas9 technology was employed. Two single guide RNAs targeting exon 53 and 53/54 intron were co‐injected with Cas9 into WT eggs. After germline transmission, the novel *dmd^−69bp^* allele was identified in which the 3′ splice site of exon 53 was removed (Figure 1D). The altered splicing of the dystrophin transcript in *dmd^−69bp^* homozygotes led to a frameshift and multiple subsequent PTCs within the dystrophin coding sequence and loss of dystrophin protein (Figure 1D,E). Myofibre retraction within *dmd^−69bp^* homozygotes was confirmed in the double transgenic background of *Tg(acta1:mCherryCaaX)* and *Tg(acta1:lifeact‐GFP)* (Figure 1F).

In summary, two novel dystrophin‐deficient mutants *dmd^pc3^* and *dmd^−69bp^* were generated: *dmd^pc3^* with the PTC UAG and *dmd^−69bp^* with a deletion not susceptible to PTC readthrough. Both mutants phenotypically match previously obtained dystrophin mutants, while their siblings remain phenotypically unremarkable.

### 3.2 Ataluren treatment over 2 days does not significantly affect the muscle integrity of dystrophin‐deficient zebrafish mutants

**FIGURE 2:**
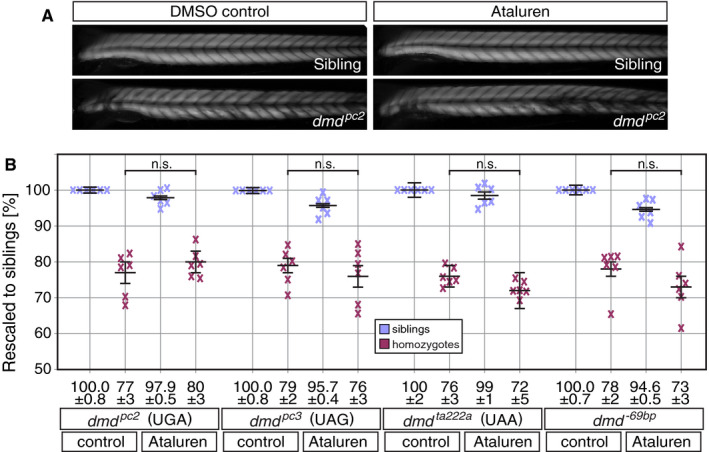
Ataluren treatment over 2 d does not have a significant effect on the amount of myofibril within dystrophin‐deficient mutants. A, Whereas 3‐dpf‐old siblings showed a uniform birefringence pattern, the stochastic myofibre degeneration within the dystrophic muscle of dystrophin‐deficient *dmd^pc2^* larvae results in a patchy birefringence pattern and an overall reduced birefringence. An effect of Ataluren treatment on the birefringence of *dmd^pc2^* homozygotes and siblings was not readily notable. B, After rescaling to DMSO‐control treated siblings, birefringence quantification revealed that 2‐d Ataluren treatment did not significantly affect the amount of muscle of homozygotes and siblings of *dmd^pc2^ dmd^pc3^*, *dmd^ta222a^* and *dmd^−69bp^*. Crosses represent averaged grey values of at least five 72‐hpf‐old larvae. Black bars represent mean ± SEM. Significance was determined by one‐way ANOVA with Tukey's multiple comparisons post hoc test, (n = 6)

To analyse the effect of Ataluren on different PTCs in an animal model of Duchenne MD, the dystrophin‐deficient mutants *dmd^pc2^*, *dmd^pc3^* and *dmd^ta222a^* (featuring UGA, UAG and UAA, respectively) and the frameshift mutant *dmd^−69bp^* were subjected to Ataluren and DMSO‐control treatment. At 24 hours after fertilization (hpf), 50 embryos per clutches were dechorionated. A total of 25 of those were exposed to 0.5 µmol/L Ataluren, the optimal concentration of Ataluren for zebrafish,^19^ and 25 were treated with DMSO vehicle control. At 72 hpf, 5 siblings and 5 random homozygotes of the DMSO‐control group and all 25 Ataluren‐treated larvae were subjected to the birefringence assay. In this procedure, individual larvae were automatically imaged under polarized light resulting in an unbiased greyscale image, in which the brightness of the imaged larvae directly represents the level of birefringence (Figure 2A). Subsequently, all larvae were genotyped and only clutches with a minimum of 5 larvae in each of the four treatment groups were analysed, namely DMSO‐treated siblings, Ataluren‐treated siblings, DMSO‐treated homozygotes and Ataluren‐treated homozygotes. The mean grey value of the first 20 somites of individual imaged larvae was measured, and all mean grey values of the same treatment group within each clutch were averaged. Thereby, per treatment and genotype at least 5 biological replicates were averaged into one grey value. Finally, to enable direct comparison of grey values, all values of each clutch were rescaled to the DMSO‐treated sibling group, which was set to 100% (Figure 2B). In total, the effect of Ataluren treatment was analysed for 6 clutches for each of the 4 different dystrophin‐deficient *dmd* lines *dmd^pc2^*, *dmd^pc3^*, *dmd^ta222a^* and *dmd^−69bp^*. Statistical analysis using one‐way ANOVA with Tukey's post hoc test revealed that Ataluren treatment over 2 days did not significantly ameliorate the dystrophic condition of dystrophin‐deficient zebrafish larvae.

In summary, a significant effect of 2‐day Ataluren treatment on dystrophin‐deficient zebrafish was not detected by extensive birefringence analysis and the reported inverse correlation of Ataluren efficacy with termination efficiencies of PTCs was not confirmed in an animal model of Duchenne MD.

### 3.3 Ataluren treatment over 5 days significantly ameliorates only *dmd^ta222a^* homozygotes that feature a nonsense mutation in exon 4 of dystrophin

**FIGURE 3:**
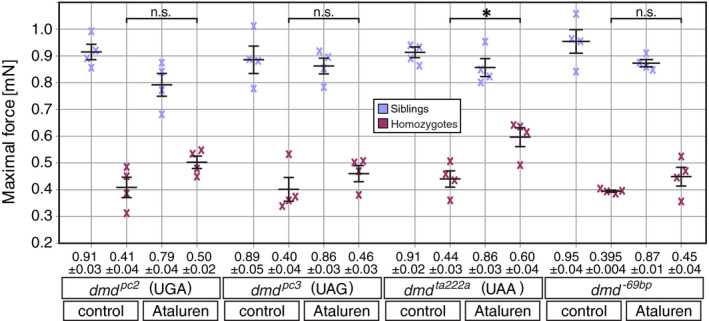
Ataluren treatment over 5 days only significantly ameliorates the force generated by *dmd^ta222a^* mutants. Direct quantification of the maximal force generated by dystrophin‐deficient larvae revealed that only the force generated by *dmd^ta222a^* homozygotes is significantly improved by 5‐d Ataluren treatment. Crosses represent force measurements of individual 6‐dpf‐old larvae. Black bars represent mean ± SEM and n.s. non‐significant. Significance was determined by one‐way ANOVA with Tukey's multiple comparisons post hoc test (*P < 0.05, n = 4)

Due to the growing muscle thickness, the birefringence assay has to be employed at early larvae stages. In order to assess the effect of Ataluren treatment over 5 days and analyse muscle function in addition to the muscle pathology at 3 dpf, the maximal force generated by 6‐dpf‐old larvae was measured. At 24 hpf, zebrafish embryos were dechorionated and exposed to either 0.5 µmol/L Ataluren or DMSO vehicle control. Solutions were renewed on a daily basis, and larvae were separated according to their phenotype at 3 dpf. At 6 dpf, 4 siblings and 4 homozygotes of each treatment group were randomly selected and subjected to force measurement (Figure 3). Subsequently, the genotype of all larvae was verified by PCR‐based genotyping. Maximal force measurements using a force transducer revealed that Ataluren treatment significantly improved the maximal force generation of homozygotes *dmd^ta222a^* larvae compared with non‐treated homozygotes (Figure 3), which was consistent with reported results demonstrating that Ataluren treatment ameliorated force generation and restored dystrophin expression in *dmd^ta222a^* homozygotes.^19^ Importantly however, the maximal force generated by homozygotes of all of the other tested mutant lines, *dmd^pc2^*, *dmd^pc3^* and *dmd^−69bp^*, was not significantly higher after 5‐day Ataluren exposure compared with non‐treated homozygotes. Thus, the only mutant that showed a beneficial effect from Ataluren treatment were *dmd^ta222a^* homozygotes that feature the PTC UAA, the type of PTC that has been reported to be least susceptible to Ataluren treatment.^3^ This might indicate that Ataluren does not ameliorate *dmd^ta222a^* homozygotes by ribosomal read‐through of PTCs.

### 3.4 Ataluren treatment has a mild, but significantly adverse effect on zebrafish

**FIGURE 4:**
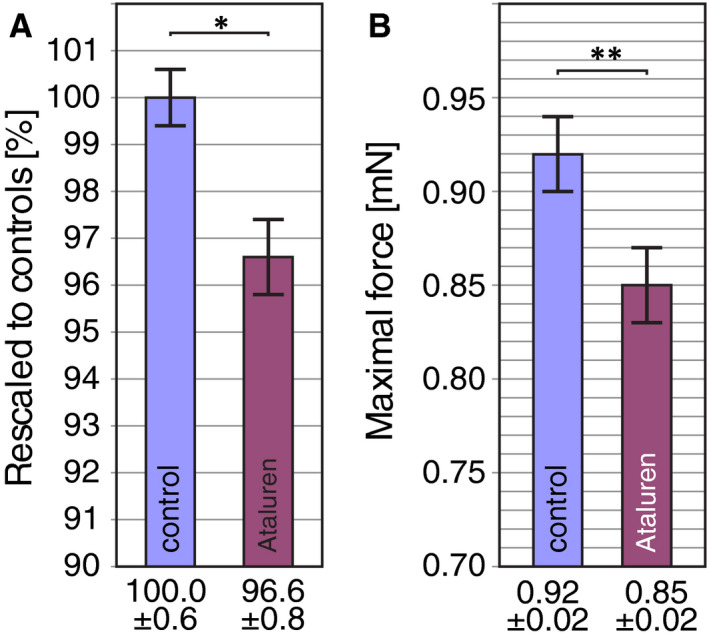
Ataluren treatment adversely affects wild‐type musculature. A, Ataluren treatment over 2 d reduced the birefringence of 3‐dpf‐old wild‐type larvae compared with DMSO‐control treated larvae. After rescaling, the birefringence of Ataluren‐treated larvae was reduced to 96.6 ± 0.8% of the birefringence of DMSO‐treated control larvae. Black bars represent mean ± SEM. Significance was determined by Student's *t* test (*P < 0.05; n = 42 for DMSO‐treated group and n = 155 for Ataluren‐treated group). B, The maximal force generated by 6‐dpf‐old WT larvae was significantly reduced in the Ataluren‐treated group (0.85 ± 0.02 mN) compared with the DMSO‐treated group (0.92 ± 0.02 mN). Black bars represent mean ± SEM. Significance was determined by Student's *t* test (**P < 0.01; n = 6 for DMSO‐treated group and n = 5 Ataluren‐treated group)

Interestingly, assessment of Ataluren treatment of four different dystrophin‐deficient zebrafish lines revealed that administration of 0.5 µmol/L Ataluren had a mild adverse effect on all tested sibling groups, although this tendency was not significant. However, by pooling the results obtained from all wild‐type (WT) larvae from the four analysed dystrophin‐deficient lines, the effect of Ataluren becomes significant (Figure 4). The birefringence of 3‐dpf‐old WT larvae that were treated with 0.5 µmol/L Ataluren over 2 days was significantly reduced in comparison to DMSO‐control WT larvae, demonstrating that Ataluren leads to a reduction in the amount of myofibril (Figure 4A). Similarly, the maximal force generated by 6‐dpf‐old WT larvae was significantly reduced after 5 days of 0.5 µmol/L Ataluren treatment compared with DMO‐treated WT controls (Figure 4B).

This result revealed that Ataluren had a mild, but significant adverse effect on the musculature of healthy wild‐type zebrafish larvae.

### 3.5 Dystrophin Transcript levels are not affected by Ataluren treatment

**FIGURE 5:**
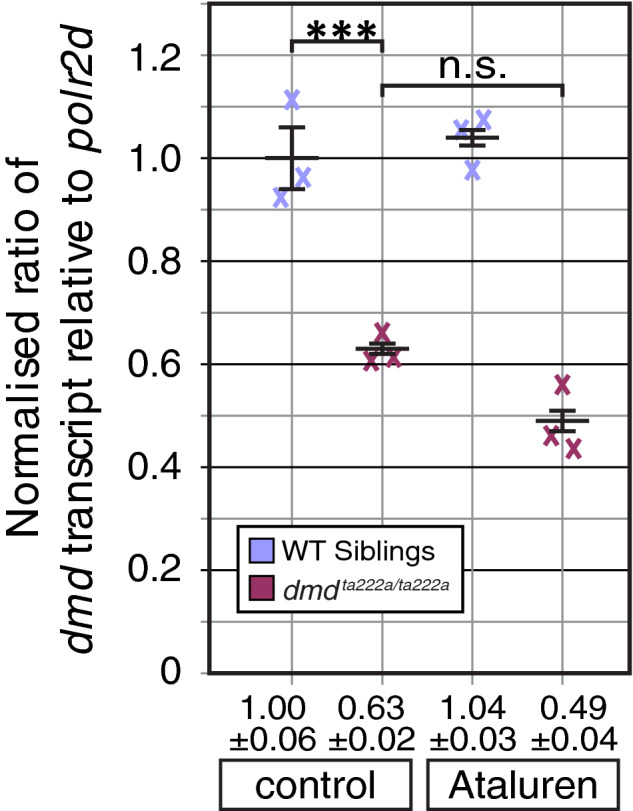
Dystrophin transcript levels are not affected by Ataluren. In triplicate ddPCR experiments, the level of *dmd* in relation to *polr2d* transcript was determined and normalized to DMSO control‐treated wild‐type siblings. Crosses represent ddPCR values, black bars mean ± SEM and n.s. non‐significant. Significance was determined by one‐way ANOVA with Tukey's multiple comparisons post hoc test (***P < 0.001, n = 3)

To assess whether the significant amelioration of the muscle force generated by *dmd^ta222a^* mutants after Ataluren treatment was based on elevated levels of dystrophin transcript, droplet digital PCR (ddPCR) was employed. Droplet digital PCR (ddPCR) has emerged as a reliable analytical technology for sequence‐specific detection and precise quantification of nucleic acids, facilitating reproducible measurement of small percentage differences even of rare variants.^30^ Similar to the force measurement assay, 24‐hpf‐old zebrafish embryos were dechorionated and exposed to either 0.5 µmol/L Ataluren or DMSO vehicle control. At 6 dpf, larvae were genotyped and two larvae per genotype (WT siblings or *dmd^ta222a^* homozygotes) and treatment group (DMSO control or Ataluren) were pooled into biological samples. Subsequently, the quantity of *dmd* transcript from three biological replicates of each of the four treatment groups was measured. In a one‐step reverse‐transcription ddPCR, dystrophin transcript levels were quantified relative to transcript levels of the *polr2d* reference gene. Compared with DMSO control‐treated wild‐type siblings, a highly significant reduction in the relative amount of *dmd* transcript was detected in control‐treated *dmd^ta222a^* homozygotes (Figure 5), likely caused by non‐sense mediated decay of mutant dystrophin transcript.^31^ However, the relative amount of *dmd* transcript in Ataluren‐treated *dmd^ta222a^* homozygotes compared with control‐treated homozygotes remained unchanged. Similarly, *dmd* transcript within wild‐type siblings was not affected by Ataluren (Figure 5).

Thereby, a significant effect of Ataluren on the level of dystrophin transcript was not detected, indicating that the effect of Ataluren on *dmd^ta222a^* might not be conveyed via dystrophin transcript.

### 3.6 N‐terminally truncated dystrophin significantly ameliorates the phenotype provoked by lack of endogenous full‐length dystrophin

Enhancement of the maximal force generated by *dmd^ta222a^* homozygotes and restoration of dystrophin protein by Ataluren has been demonstrated.^19^ Further pathological and functional assessment of the muscle of four dystrophin‐deficient mutant lines, including mutants for all three PTCs, revealed that *dmd^ta222a^* was the only mutant line ameliorated by Ataluren, indicating that Ataluren might not suppress PTCs. Interestingly, *dmd^ta222a^* mutants harbour an early nonsense mutation within exon 4, whereas all other, unaffected mutants *dmd^pc2^*, *dmd^pc3^* and *dmd^−69bp^* carry mutations located further downstream within exons 32, 34 and 53, respectively. Thereby, the possibility arises that Ataluren treatment might enhance the generation of N‐terminally truncated dystrophin protein from an alternative translation start codon (ATG) located within exons 6 and 8.

**FIGURE 6:**
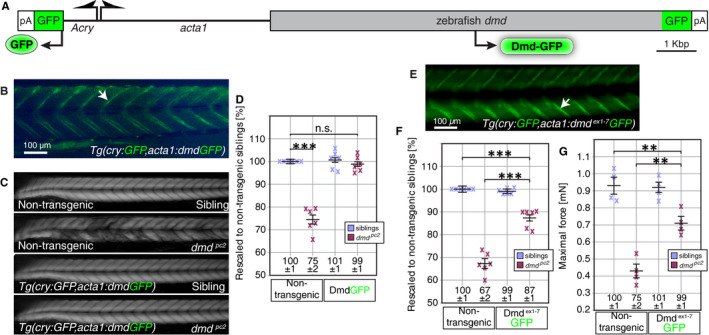
The dystrophin isoform Dmd^∆ex1‐7^ lacking exons 1‐7 is partially functional. A, Schematic of the construct used to generate the transgenic line *Tg(αAcry:GFP,acta1:dmdGFP)*. The *acta1* promoter directed full‐length dystrophin into muscle and the *αA‐crystallin* promoter (*αAcry*) drove GFP expression into the lens for rapid identification of transgenic animals. pA indicates SV40 polyA signal. B, In 3‐dpf‐old *Tg(cry:GFP,acta1:dmdGFP)* larvae, fluorescence of the Dmd‐GFP fusion protein was detected at the vertical myosepta (arrowheads). C, Polarized light visualized the muscle of 3‐dpf‐old larvae. Whereas the birefringence of non‐transgenic siblings appeared uniform, the birefringence of *dmd^pc2^* homozygotes appeared patchy. In contrast, in the transgenic background of *Tg(cry:GFP,acta1:dmdGFP)* the birefringence of *dmd^pc2^* homozygotes and siblings was comparable. D, Quantification of the birefringence followed by rescaling to non‐transgenic siblings revealed that the birefringence of *dmd^pc2^* homozygotes transgenic for *Tg(cry:GFP,acta1:dmdGFP)* was not significantly changed compared with non‐transgenic *dmd^pc2^* siblings. Crosses represent averaged grey values of at least five 72‐hpf‐old larvae. Black bars represent mean ± SEM and n.s. non‐significant. Significance was determined by one‐way ANOVA with Tukey's multiple comparisons post hoc test (***P < 0.001, n = 6). E, Within *Tg(cry:GFP,acta1:dmd^∆ex1‐7^GFP)* larvae, N‐terminally truncated dystrophin fused to GFP localized to the vertical myosepta as indicated by the GFP fluorescence. F, After rescaling to non‐transgenic siblings, the birefringence of *dmd^pc2^* homozygotes transgenic for *Tg(cry:GFP,acta1:dmd^∆ex1‐7^GFP)* was significantly ameliorated compared with non‐transgenic *dmd^pc2^* homozygotes, but significantly reduced compared with non‐transgenic *dmd^pc2^* siblings. Crosses represent averaged grey values of at least five 72‐hpf‐old larvae. Black bars are mean ± SEM Significance was determined by one‐way ANOVA with Tukey's multiple comparisons post hoc test (***P < 0.001, n = 6). G, At 6 dpf, the maximal force generated by *dmd^pc2^* homozygotes positive for *Tg(cry:GFP,acta1:dmd^∆ex1‐7^GFP)* was significantly stronger compared with non‐transgenic *dmd^pc2^* homozygotes, but significantly reduced compared with non‐transgenic *dmd^pc2^* siblings. Crosses represent individual larvae and black bars mean ± SEM. Significance was determined by one‐way ANOVA with Tukey's multiple comparisons post hoc test (**P < 0.01, n = 4)

To establish an in vivo system to analyse functionality of dystrophin lacking exons 1‐7 in vivo, rescue of dystrophin‐deficient zebrafish by transgenic full‐length dystrophin was analysed as a positive control. Dystrophin cDNA was cloned from 3‐dpf‐old WT larvae and used to generate the transgenic line *Tg(cry:GFP,acta1:dmdGFP)*. *Tg(cry:GFP,acta1:dmdGFP)* fish were fully viable, expressed the Dmd‐GFP fusion protein under the control of the muscle‐specific *acta1* promoter^27^ and directed GFP into the lens by the *αA‐crystallin* promoter for fast identification of transgenic fish^26^ (Figure 6A). Importantly, GFP fluorescence within *Tg(cry:GFP,acta1:dmdGFP)* was not only detected in the lens but also at the myotendinous junctions, indicating that Dmd‐GFP fusion protein replicated the localization of endogenous dystrophin (Figure 6B).

To analyse the rescue of the dystrophic phenotype of dystrophin mutants lacking endogenous dystrophin, *dmd^pc2^* was crossed to into the *Tg(cry:GFP,acta1:dmdGFP)* transgenic background and their birefringence was assessed at 72 hpf (Figure 6C). In contrast to non‐transgenic *dmd^pc2^* homozygotes that featured a highly significant reduction in birefringence compared with their non‐transgenic siblings, the birefringence of *dmd^pc2^* homozygotes in the *Tg(cry:GFP,acta1:dmdGFP)* background was comparable to non‐transgenic siblings. The rescue of *dmd^pc2^* homozygotes indicates that the transgenic full‐length dystrophin, fused to GFP and expressed from a transgene under the *acta1* promoter, was fully functional (Figure 6D).

In an equivalent approach, the transgenic line *Tg(cry:GFP,acta1:dmd^∆ex1‐7^GFP)* was generated with dystrophin lacking exons 1‐7. Affirmed by the GFP fluorescence, the N‐terminally truncated dystrophin fused to GFP localized to vertical myosepta in *Tg(cry:GFP,acta1:dmd^∆ex1‐7^GFP)* (Figure 6E). At 72 hpf, birefringence quantification showed that *dmd^pc2^* homozygotes in the *Tg(cry:GFP,acta1:dmd^∆ex1‐7^GFP)* background were significantly ameliorated compared with non‐transgenic *dmd^pc2^* homozygotes (Figure 6F). However, the birefringence of *dmd^pc2^* homozygotes positive for *Tg(cry:GFP,acta1:dmd^∆ex1‐7^GFP)* was still reduced compared with non‐transgenic siblings, indicating that N‐terminally truncated dystrophin only partially rescued the dystrophic phenotype of *dmd^pc2^* mutants. A similar result was observed with force measurements of 6‐dpf‐old larvae. The reduction in maximal force generated by *dmd^pc2^* homozygotes in the *Tg(cry:GFP,acta1:dmd^∆ex1‐7^GFP)* background was highly significant compared with non‐transgenic siblings, but ameliorated compared with non‐transgenic *dmd^pc2^* homozygotes, confirming the partial functionality of N‐terminally truncated dystrophin (Figure 6G).

In transgenic zebrafish, dystrophin lacking exons 1‐7 was able to ameliorate the dystrophic pathology. One could therefore speculate that Ataluren could enhance translation of dystrophin transcript from alternative start codons.

## 4 DISCUSSION

The efficacy of Ataluren remains disputed, and its mechanism of action has not been resolved utilizing animal models and cultured cells. Additionally, Ataluren assessment in patients suffering from Duchenne MD is hampered by nonsense mutations that alter exon splicing,^32^, ^33^ the broad spectrum of symptoms, the constant decline in muscle weakness, and the restriction of patients in clinical trials from established treatments. As a result, Ataluren efficacy for Duchenne MD patients is not fully established and the cohort of responsive patients has not been identified.

To improve our knowledge on the efficacy and the mechanism of action of Ataluren, a large‐scale zebrafish analysis has been performed with two robust and quantifiable analysis methods. Although the beneficial effect of Ataluren treatment over 5 days was confirmed in the dystrophin‐deficient line *dmd^ta222a^*,^19^ the muscle pathology and function of all other analysed mutant lines *dmd^pc2^*, *dmd^pc3^* and *dmd^−69bp^* was not improved by Ataluren treatment. This is of particular interest, as the PTC harboured by *dmd^ta222a^* is TAA, which has been reported to be least susceptible to Ataluren utilizing in vitro experiments.^3^ The other dystrophin‐deficient lines *dmd^pc2^*, *dmd^pc3^* harbour the weaker PTCs UAG and UGA that, based on reported data,^3^ are predicted to be more susceptible to Ataluren. These findings are consistent with small‐scale clinical studies of Ataluren, in which only a subgroup of nonsense mutation Duchenne muscular dystrophy patients benefitted and no correlation with PTC types was found.^10^, ^12^ Although PTC suppression by Ataluren has been confirmed in vitro,^34^ human trials and our zebrafish study, these studies combined indicate that additional factors might contribute to the beneficial effect of Ataluren. A potential contributing factor might be the complex influence of nucleotides downstream of the PTC that have been reported to alter the termination efficiency of PTCs.^35^

Pooling of results obtained from wild type revealed that Ataluren had mild detrimental effects on the muscle of zebrafish larvae, which showed a reduction of the amount of myofibril after 3‐day treatment and weakening of the muscle after 5‐day treatment. This finding is in agreement with results from clinical trials involving Duchenne MD patients, reporting mild to moderate adverse clinical events.^8^, ^10^, ^12^ Accordingly, emerging evidence has been brought forward that lower doses of read‐through supressing aminoglycosides increased misincorporation of amino acids during translation^36^ and that aminoglycosides induced damage to the kidney and the inner ear.^37^, ^38^ Therefore, although Ataluren was generally well tolerated,^8^, ^10^ caution is required when Ataluren is provided to patients.

To test the possibility of Ataluren enforcing translation from alternative ATG codons, the functionality of N‐terminally truncated dystrophin was assessed in transgenic animals. Partial functionality of dystrophin lacking exons 1‐7 was indicated by the partial rescue of the dystrophic muscle of *dmd^pc2^* homozygotes. These findings are consistent with patient reports revealing a nonsense mutation within exon 1 resulting in the mild Becker MD symptoms due to the alternative translation initiation at two AUG codons located in exon 6,^39^ which are employed by internal ribosomal entry site within exon 5.^40^ Similarly, for the frame‐shifting deletion of exons 3‐7 that result in Becker MD, it has been proposed that an alternative ATG in exon 8 could be used for translation initiation.^41^ These findings open the possibility of another route of mechanism for the action of Ataluren, in which alternative translation starts are employed to generate shorter but largely functional dystrophin protein. However, whether truncated dystrophin protein was generated in *dmd^ta222a^* homozygotes after Ataluren treatment has not been assessed directly.

Duchenne MD trials and our zebrafish study indicate that the mechanism of action of Ataluren has not fully been uncovered. Our additional results from transgenic zebrafish opened the possibility that Ataluren might contribute by promoting dystrophin translation from alternative translation start codons. However, further insights are required to fully establish the mechanism of action of Ataluren in order to identify the cohort of patients responsive to Ataluren.

## CONFLICT OF INTEREST

The authors confirm that there are no conflicts of interest.

## AUTHOR CONTRIBUTIONS

JB conceived the study and performed experiments. ML and SB performed force measurements. MM and JR performed ddPCR. JB and PDC wrote the manuscript, which was approved by all authors.

## Data Availability

Raw data can be obtained from the corresponding authors on request.

## References

[1] McDonald CM , Henricson EK , Abresch RT , et al. Long‐term effects of glucocorticoids on function, quality of life, and survival in patients with Duchenne muscular dystrophy: a prospective cohort study. Lancet. 2018;391:451‐461. 29174484 10.1016/S0140-6736(17)32160-8

[2] Khan N , Eliopoulos H , Han L , et al. Eteplirsen treatment attenuates respiratory decline in ambulatory and non‐ambulatory patients with duchenne muscular dystrophy. J Neuromuscul Dis. 2019;6:213‐225. 30856119 10.3233/JND-180351 PMC6598025

[3] Welch EM , Barton ER , Zhuo J , et al. PTC124 targets genetic disorders caused by nonsense mutations. Nature. 2007;447:87‐91. 17450125 10.1038/nature05756

[4] Auld DS , Thorne N , Maguire WF , Inglese J . Mechanism of PTC124 activity in cell‐based luciferase assays of nonsense codon suppression. Proc Natl Acad Sci USA. 2009;106:3585‐3590. 19208811 10.1073/pnas.0813345106 PMC2638738

[5] Auld DS , Lovell S , Thorne N , et al. Molecular basis for the high‐affinity binding and stabilization of firefly luciferase by PTC124. Proc Natl Acad Sci USA. 2010;107:4878‐4883. 20194791 10.1073/pnas.0909141107 PMC2841876

[6] Pichavant C , Aartsma‐Rus A , Clemens PR , et al. Current status of pharmaceutical and genetic therapeutic approaches to treat DMD. Mol Ther. 2011;19:830‐840. 21468001 10.1038/mt.2011.59 PMC3098643

[7] Laing NG , Davis MR , Bayley K , Fletcher S , Wilton SD . Molecular diagnosis of duchenne muscular dystrophy: past, present and future in relation to implementing therapies. Clin Biochem Rev. 2011;32:129‐134. 21912442 PMC3157948

[8] Ebrahimi‐Fakhari D , Dillmann U , Flotats‐Bastardas M , et al. Off‐label use of ataluren in four non‐ambulatory patients with nonsense mutation duchenne muscular dystrophy: effects on cardiac and pulmonary function and muscle strength. Front Pediatr. 2018;6:316. 30406066 10.3389/fped.2018.00316 PMC6206203

[9] Bushby K , Finkel R , Wong B , et al. Ptc124‐Gd‐007‐Dmd Study G. Ataluren treatment of patients with nonsense mutation dystrophinopathy. Muscle Nerve. 2014;50:477‐487. 25042182 10.1002/mus.24332 PMC4241581

[10] McDonald CM , Campbell C , Torricelli RE , et al. Clinical evaluator training G, group ADS. Ataluren in patients with nonsense mutation Duchenne muscular dystrophy (ACT DMD): a multicentre, randomised, double‐blind, placebo‐controlled, phase 3 trial. Lancet. 2017;390:1489‐1498. 28728956 10.1016/S0140-6736(17)31611-2

[11] Mercuri E , Muntoni F , Osorio AN , et al. Safety and effectiveness of ataluren: comparison of results from the STRIDE Registry and CINRG DMD Natural History Study. J Comp Eff Res. 2020 10.2217/cer-2019-0171 PMC7610147 31997646

[12] Finkel RS , Flanigan KM , Wong B , et al. Phase 2a study of ataluren‐mediated dystrophin production in patients with nonsense mutation Duchenne muscular dystrophy. PLoS ONE. 2013;8:e81302. 24349052 10.1371/journal.pone.0081302 PMC3859499

[13] Haas M , Vlcek V , Balabanov P , et al. European Medicines Agency review of ataluren for the treatment of ambulant patients aged 5 years and older with Duchenne muscular dystrophy resulting from a nonsense mutation in the dystrophin gene. Neuromuscul Disord. 2015;25:5‐13. 25497400 10.1016/j.nmd.2014.11.011

[14] Berger J , Berger S , Hall TE , Lieschke GJ , Currie PD . Dystrophin‐deficient zebrafish feature aspects of the Duchenne muscular dystrophy pathology. Neuromuscul Disord. 2010;20:826‐832. 20850317 10.1016/j.nmd.2010.08.004

[15] Berger J , Sztal T , Currie PD . Quantification of birefringence readily measures the level of muscle damage in zebrafish. Biochem Biophys Res Comm. 2012;423:785‐788. 22713473 10.1016/j.bbrc.2012.06.040

[16] Dou Y , Andersson‐Lendahl M , Arner A . Structure and function of skeletal muscle in zebrafish early larvae. J Gen Physiol. 2008;131:445‐453. 18443359 10.1085/jgp.200809982 PMC2346565

[17] Dowling JJ , Arbogast S , Hur J , et al. Oxidative stress and successful antioxidant treatment in models of RYR1‐related myopathy. Brain. 2012;135:1115‐1127. 22418739 10.1093/brain/aws036 PMC3326256

[18] Berger J , Berger S , Li M , Currie PD . Myo18b is essential for sarcomere assembly in fast skeletal muscle. Hum Mol Genet. 2017;26:1146‐1156. 28104788 10.1093/hmg/ddx025

[19] Li M , Andersson‐Lendahl M , Sejersen T , Arner A . Muscle dysfunction and structural defects of dystrophin‐null sapje mutant zebrafish larvae are rescued by ataluren treatment. FASEB J. 2014;28:1593‐1599. 24371125 10.1096/fj.13-240044

[20] Berger J , Berger S , Jacoby AS , Wilton SD , Currie PD . Evaluation of exon‐skipping strategies for Duchenne muscular dystrophy utilizing dystrophin‐deficient zebrafish. J Cell Mol Med. 2011;15:2643‐2651. 21251213 10.1111/j.1582-4934.2011.01260.x PMC4373433

[21] Berger J , Berger S , Li M , et al. In vivo function of the chaperonin TRiC in alpha‐actin folding during sarcomere assembly. Cell Rep. 2018;22:313‐322. 29320728 10.1016/j.celrep.2017.12.069

[22] Kimmel CB , Ballard WW , Kimmel SR , Ullmann B , Schilling TF . Stages of embryonic development of the zebrafish. Dev Dyn. 1995;203:253‐310. 8589427 10.1002/aja.1002030302

[23] Li M , Andersson‐Lendahl M , Sejersen T , Arner A . Knockdown of desmin in zebrafish larvae affects interfilament spacing and mechanical properties of skeletal muscle. J Gen Physiol. 2013;141:335‐345. 23440276 10.1085/jgp.201210915 PMC3581687

[24] Lai D , Lan CC , Leong IU , Love DR . Zebrafish dystrophin and utrophin genes: dissecting transcriptional expression during embryonic development. Int J Mol Med. 2012;29:338‐348. 22200618 10.3892/ijmm.2011.865

[25] Jacoby AS , Busch‐Nentwich E , Bryson‐Richardson RJ , et al. The zebrafish dystrophic mutant softy maintains muscle fibre viability despite basement membrane rupture and muscle detachment. Development. 2009;136:3367‐3376. 19736328 10.1242/dev.034561 PMC2739150

[26] Berger J , Currie PD . 503unc, a small and muscle‐specific zebrafish promoter. Genesis. 2013;51:443‐447. 23444339 10.1002/dvg.22385

[27] Higashijima S , Okamoto H , Ueno N , Hotta Y , Eguchi G . High‐frequency generation of transgenic zebrafish which reliably express GFP in whole muscles or the whole body by using promoters of zebrafish origin. Dev Biol. 1997;192:289‐299. 9441668 10.1006/dbio.1997.8779

[28] Bassett DI , Bryson‐Richardson RJ , Daggett DF , Gautier P , Keenan DG , Currie PD . Dystrophin is required for the formation of stable muscle attachments in the zebrafish embryo. Development. 2003;130:5851‐5860. 14573513 10.1242/dev.00799

[29] Berger J , Tarakci H , Berger S , et al. Loss of Tropomodulin4 in the zebrafish mutant trage causes cytoplasmic rod formation and muscle weakness reminiscent of nemaline myopathy. Dis Model Mech. 2014;7:1407‐1415. 25288681 10.1242/dmm.017376 PMC4257009

[30] Taylor SC , Laperriere G , Germain H . Droplet Digital PCR versus qPCR for gene expression analysis with low abundant targets: from variable nonsense to publication quality data. Sci Rep. 2017;7:2409. 28546538 10.1038/s41598-017-02217-x PMC5445070

[31] Amrani N , Sachs MS , Jacobson A . Early nonsense: mRNA decay solves a translational problem. Nat Rev Mol Cell Biol. 2006;7:415‐425. 16723977 10.1038/nrm1942

[32] Ginjaar IB , Kneppers ALJ , v d Meulen J‐D , et al. Dystrophin nonsense mutation induces different levels of exon 29 skipping and leads to variable phenotypes within one BMD family. Eur J Hum Genet. 2000;8:793‐796. 11039581 10.1038/sj.ejhg.5200535

[33] Flanigan KM , Dunn DM , von Niederhausern A , et al. Nonsense mutation‐associated Becker muscular dystrophy: interplay between exon definition and splicing regulatory elements within the DMD gene. Hum Mutat. 2011;32:299‐308. 21972111 10.1002/humu.21426 PMC3724403

[34] Roy B , Friesen WJ , Tomizawa Y , et al. Ataluren stimulates ribosomal selection of near‐cognate tRNAs to promote nonsense suppression. Proc Natl Acad Sci USA. 2016;113:12508‐12513. 27702906 10.1073/pnas.1605336113 PMC5098639

[35] Cridge AG , Crowe‐McAuliffe C , Mathew SF , Tate WP . Eukaryotic translational termination efficiency is influenced by the 3' nucleotides within the ribosomal mRNA channel. Nucleic Acids Res. 2018;46:1927‐1944. 29325104 10.1093/nar/gkx1315 PMC5829715

[36] Keeling KM , Wang D , Conard SE , Bedwell DM . Suppression of premature termination codons as a therapeutic approach. Crit Rev Biochem Mol Biol. 2012;47:444‐463. 22672057 10.3109/10409238.2012.694846 PMC3432268

[37] Huth ME , Ricci AJ , Cheng AG . Mechanisms of aminoglycoside ototoxicity and targets of hair cell protection. Int J Otolaryngol. 2011;2011:937861. 22121370 10.1155/2011/937861 PMC3202092

[38] Lopez‐Novoa JM , Quiros Y , Vicente L , Morales AI , Lopez‐Hernandez FJ . New insights into the mechanism of aminoglycoside nephrotoxicity: an integrative point of view. Kidney Int. 2011;79:33‐45. 20861826 10.1038/ki.2010.337

[39] Gurvich OL , Maiti B , Weiss RB , Aggarwal G , Howard MT , Flanigan KM . DMD exon 1 truncating point mutations: amelioration of phenotype by alternative translation initiation in exon 6. Hum Mutat. 2009;30:633‐640. 19206170 10.1002/humu.20913 PMC2663021

[40] Wein N , Vulin A , Falzarano MS , et al. Translation from a DMD exon 5 IRES results in a functional dystrophin isoform that attenuates dystrophinopathy in humans and mice. Nat Med. 2014;20:992‐1000. 25108525 10.1038/nm.3628 PMC4165597

[41] Winnard AV , Mendell JR , Prior TW , Florence J , Burghes AH . Frameshift deletions of exons 3–7 and revertant fibers in Duchenne muscular dystrophy: mechanisms of dystrophin production. Am J Hum Genet. 1995;56:158‐166. 7825572 PMC1801338

